# Overlap in signaling between Smoothened and the α subunit of the heterotrimeric G protein G_13_

**DOI:** 10.1371/journal.pone.0197442

**Published:** 2018-05-15

**Authors:** Xueshui Guo, Natalia A. Riobo-Del Galdo, Eun Ji Kim, Gregory R. Grant, David R. Manning

**Affiliations:** 1 Department of Systems Pharmacology and Translational Therapeutics, Perelman School of Medicine, University of Pennsylvania, Philadelphia, Pennsylvania, United States of America; 2 Leeds Institute of Cancer and Pathology and School of Molecular and Cellular Biology, University of Leeds, United Kingdom; 3 Institute of Translational Medicine and Therapeutics, Perelman School of Medicine, University of Pennsylvania, Philadelphia, Pennsylvania, United States of America; 4 Department of Genetics, Perelman School of Medicine, University of Pennsylvania, Philadelphia, Pennsylvania, United States of America; University of Navarra, SPAIN

## Abstract

The Hedgehog family of morphogens has long been known to utilize, through the 7-transmembrane protein Smoothened (Smo), the heterotrimeric G protein G_i_ in both canonical and noncanonical forms of signaling. Other G proteins, while not specifically utilized by Smo, may nonetheless provide access to some of the events controlled by it. We reported several years ago that the G protein G_13_ activates one or more forms of the Gli family of transcription factors. While the Gli transcription factors are well known targets for Smo, the uncertain mechanism of activation by G_13_ and the identity of the targeted Gli(s) limited predictions as to the extent to which G_13_ might mimic Smo’s actions. We evaluate here the potential for overlap in G_13_ and Smo signaling using C3H10T1/2 and 3T3-L1 cells as models of osteogenesis and adipogenesis, respectively. We find in C3H10T1/2 cells that a constitutively active form of Gα_13_ (Gα_13_QL) increases Gli1 mRNA, as does a constitutively active form of Smo (SmoA1). We find as well that Gα_13_QL induces alkaline phosphatase activity, a marker of osteogenesis, albeit the induction is far less substantial than that achieved by SmoA1. In 3T3-L1 cells both Gα_13_QL and SmoA1 markedly suppress adipogenic differentiation as determined by triglyceride accumulation. RNA sequencing reveals that Gα_13_QL and SmoA1 regulate many of the same genes but that quantitative and qualitative differences exist. Differences also exist, we find, between SmoA1 and purmorphamine, an agonist for Smo. Therefore, while comparisons of constitutively active proteins are informative, extrapolations to the setting of agonists require care.

## Introduction

Considerable interest exists in the extent to which members of the Hedgehog family of secreted morphogens utilize heterotrimeric G proteins. The interest stems, first, from the fact that the signaling initiated by Hedgehogs is largely directed through Smoothened (Smo), a protein that exhibits a 7-transmembrane (7-TM) motif common to receptors that interact with G proteins [1,2]. Second, certain actions of Hedgehogs (through Smo) are sensitive to a pertussis toxin known to disrupt signaling through the G protein G_i_ [3–7]. Third, these and certain other [8,9] actions of Hedgehogs occur more rapidly than can be easily accommodated by ‘canonical’ signaling. Canonical signaling refers to the sequence of events that begins with the activation of Smo and culminates in the stabilization of Gli2 and Gli3 and consequent expression of Gli1 zinc-finger transcription factors (Gli) [10,11]. Canonical signaling sometimes requires G_i_, but not always [3,12–14]. Finally, Smo is capable of coupling to all members of the G_i_ family (i.e. G_i_, G_o_, and G_z_) and, as determined by GTP/GDP exchange, couples to this family alone with a strength comparable to other G_i_-coupled receptors [12,15].

Given the selectivity of Smo for G_i_ and what seems to be the mostly dedicated control of Gli transcription factors by Smo, we were surprised to discover that G proteins beyond G_i_ could activate reporter genes for Gli [16]. The G protein exhibiting the greatest activity in this regard was G_13_. Both wildtype and an active mutant of the α subunit of G_13_ (Gα_13_) increased the activity of Gli reporters in mouse C3H10T1/2 and human pancreatic cancer cells. This action was replicated in the latter cells by the G_q_- and G_12/13_-coupled cholecystokinin-A receptor and, as an agonist for this receptor, the C-terminal octapeptide of cholecystokinin. Cyclopamine, an antagonist at the level of Smo, had no effect on the actions of Gα_13_QL, precluding an indirect, autocrine activation. We proposed that G proteins such as G_13_ can provide access to Gli transcription factors apart from that provided by Smo.

We became interested, therefore, in the question of convergent signaling through Smo and G_13_, specifically the degree to which G_13_ can replicate the actions achieved by Smo through or apart from Gli transcription factors. The shared use of one or more of these transcription factors provides an inducement for this work, of course, but Smo and G_13_ have many other actions concordant with development [17–22]. The degree of overlap would help in understanding the extent to which agonists working through G_13_ might engage phenomena otherwise controlled by Hedgehogs. Here, using C3H10T1/2 and NIH 3T3-L1 cells as models for transition of mesenchymal-like cells toward osteoblastic and adipogenic lineages, respectively, and using recombinant adenoviruses in a modified transduction paradigm, we evaluated the actions of constitutively active forms of Smo and Gα_13_. We found that these two proteins share many actions in common, however differences clearly exist. We also found considerable differences to exist between constitutively active Smo and the Smo agonist purmorphamine, suggesting that extrapolation to the setting of an agonist can be complicated by what appear to be biases in signaling at one or more levels.

## Materials and methods

### Materials

Mouse C3H10T1/2 (CCL-226) and 3T3-L1 (CL-173) cells were obtained directly from American Type Culture Collection (Manassas, VA). Mouse SmoA1 cDNA [23] was a gift from Dr. Philip Beachy (Stanford University, CA). Human Gα_13_QL cDNA was provided by the cDNA Resource Center (www.cdna.org). pShuttle-CMV-lacZ and -GFP were obtained from Agilent Technologies (Santa Clara, CA). Polybrene, DEAE-dextran, and purmorphamine were obtained from Sigma-Aldrich (St. Louis, MO).

### Recombinant adenovirus constructs

Construction of recombinant adenoviruses was accomplished with reagents and instructions provided by Agilent Technologies. SmoA1 and Gα_13_QL were introduced into pShuttle-CMV by subcloning. pShuttle-CMV-LacZ, -GFP, -SmoA1, and -Gα_13_QL, respectively, were linearized and cotransformed with pAdEasy into BJ5183 by electroporation. Homologous recombinants were transformed into XL-10. Recombinants were PacI-digested and transfected into AD-293 cells to obtain primary viral stocks. Primary and subsequent stocks were amplified (up to 5 rounds total) as needed using AD-293 cells. Viral titers were determined using instructions provided with the AdEasy Viral Titer Kit (Agilent Technologies). Amplified stocks generally contained 5–15 x 10^8^ infectious units per ml. The recombinant viruses are referred to as AdV·LacZ, AdV·GFP, AdV·SmoA1, and AdV·Gα_13_QL.

### Cell culture and transformation

C3H10T1/2 cells were maintained in Basal Medium Eagle (BME) supplemented with 2 mM L-glutamine and 10% fetal bovine serum (FBS) at 37°C at 5% CO_2_; we did not use cells beyond 12 passages from purchase. 3T3-L1 cells were maintained in Dulbecco’s Modified Eagle Medium (DMEM) with 10% FBS at 37°C at 5% CO_2_. For most experiments involving transformation, cells at 80–90% confluence were incubated in medium without serum for 12 h then with recombinant adenovirus at the specified multiplicity of infection (MOI, i.e. infectious units per cell) in medium with 1% FBS for 6 h, with or without polycations as described in individual experiments. Cells destined for PCR or RNA-seq were transferred to fresh medium with 1% FBS for another 42 h; cells destined for alkaline phosphatase or Oil Red O assays were processed as described below. For experiments relating to stress fiber formation, cells at 60–70% were incubated with recombinant adenovirus for 6 h in medium without serum, then transferred to fresh medium, again without serum, for 18 h. Experiments with AdV·LacZ, AdV·SmoA1, and AdV·Gα_13_QL beyond those relating to optimization were most often performed at an MOI of 10 based on initial measurements of titers. Reevaluation of titers at the culmination of the experiments revealed MOIs of 8, 10, and 14 respectively. We retain the use of 10 as a nominal MOI in descriptions of the experiments.

### Assay for LacZ activity

LacZ activity was evaluated using chlorophenol red-β-D-galactopyranoside as described for the High Sensitivity β-galactosidase Assay Kit (Agilent Technologies). Cell lysates were incubated with the substrate usually for 30 min at room temperature. Absorbance was measured at 595 nm.

### Detection of Gli1 RNA by polymerase chain reaction

RNA was extracted using the RNeasy Mini Kit from QIAGEN (Valencia, CA). Reverse transcription was achieved using TaqMan Reverse Transcription Reagents from Life Technologies (Carlsbad, CA). PCR was performed using Taq DNA polymerase from Sigma using, for Gli1, 5’CCTAGCAATAAGGCTCCGGG3’ as the forward primer and 5’GGTTGGTATCCCCCACCTTG3’ as the reverse primer and, for GAPDH, 5’GTGGCAAAGTGGAGATTG3’ as the forward primer and 5’TCAGTGTAGCCCAAGATG3’ as the reverse primer. Denaturation, annealing, and chain elongation were performed at 94°, 60°, and 72°C, respectively, usually for 30 cycles.

### Phalloidin staining

Cells were fixed with 4% paraformaldehyde, blocked with bovine serum albumin, permeabilized with 0.1% Triton X-100, and incubated with 5 μg/ml fluorescein isothiocyanate (FITC)-Phalloidin from Sigma [24,25]. Cells were counterstained with 1 μg/ml 4’,6-diamidino-2-phenylindole (DAPI). Coverslips were mounted in VECTASHIELD Antifade Mounting Medium from Vector Labs (Burlingame, CA). A Zeiss-Axiophot microscope (40X objective, with an additional 1.6X magnification) was used to identify positive cells, which were counted in 12–20 random fields for each treatment. Exposure times were 400 ms and 20 μs for FITC and DAPI, respectively.

### Alkaline phosphatase expression

Cells infected with recombinant adenoviruses, or treated with purmorphamine as noted, were grown in BME supplemented with 2 mM glutamine, 50 μg/ml ascorbic acid, 10 mM β-glycerophosphate, and 10% FBS for 7–8 days. Cells were solubilized in 150 mM NaCl, 5 mM ethylendiaminetetraacetic acid, pH 8.0, 50 mM Tris, pH 8.0, 1% Nonidet P-40, 0.5% sodium deoxycholate, and 0.1% sodium dodecyl sulfate and cleared by centrifugation. Each cell extract (20 μl) was combined with 160 μl 1 M diethanolamine plus 0.5 mM MgCl_2_, pH 9.8, and 20 μl of 1 mg/ml *p*-nitrophenyl phosphate in one well of a 96-well plate. The plate was incubated at 37° C for 20–60 min, at which time absorbance at 400 nm was measured.

### Adipogenic differentiation and Oil Red O staining

We followed the adipogenic differentiation protocol devised by Zebisch et al [26]. 3T3-L1 cells infected with recombinant adenoviruses were cultured for 3 days in DMEM containing 10% FBS, then cultured for 2 days in the same medium but containing as well 0.5 mM isobutylmethylxanthine, 0.25 μM dexamethasone, 1 μg/ml insulin, and 2 μM rosiglitazone for 48 h, then for 2 days in the same medium but with insulin alone, then for one week in the original medium without supplementation. Oil Red O staining was evaluated with a protocol provided by Lonza (Walkersville, MD). Briefly, the cells were fixed with 10% formalin then incubated with Oil Red O (Sigma); affixed Oil Red O was extracted with isopropanol and quantitated by absorbance at 490 nm.

### Purmorphamine treatment prior to RNA-sequencing

C3H10T1/2 cells were grown to 85% confluency, serum-starved in medium without FBS for 12 hr, then treated with 2 μM purmorphamine or vehicle (dimethylsufoxide (DMSO), 0.02%) for 48 h in the continued absence of serum. The concentration of 2 μM was found to be optimal in assays of alkaline phosphatase expression (S1 Fig).

### RNA-sequencing

Cells were harvested two days following transduction with recombinant adenoviruses or exposure to purmorphamine for total RNA extraction using the RNeasy Minikit (QIAGEN). RNA quality (RNA integrity number > 8) was assured by analysis with an Agilent Bioanalyzer and an Invitrogen Qubit fluorometric assay. RNA-sequencing (RNA-seq) was performed by the Next Generation Sequencing Core at the Penn campus using a TruSeq Stranded Total RNA Library Prep Kit with Ribo-Zero Gold. Single-read sequencing was performed on an Illumina HiSeq 4000 to 100 bases. Data were normalized using a read resampling approach to equalize the read counts across all samples before quantifying (https://github.com/itmat/Normalization). Data were quantified at the gene level and (FDR-based) q-values for differentially expressed genes were obtained using Limma-Voom followed by Benjamini-Hochberg. Genes were sorted by q-value. Data are available through GEO (GSE98841, for proteins introduced by means of recombinant adenoviruses, and GSE111669, for purmorphamine). Fold increases or decreases for transcripts in response to SmoA1 and Gα_13_QL relative to LacZ, or of purmorphamine relative to vehicle (DMSO), were determined based on normalized reads; in the case of -fold increases with SmoA1 or Gα_13_QL, or with purmorphamine, for any gene whose expression could not be detected with LacZ, or with vehicle in the case of purmorphamine, we used a nominal value of 1 read for the divisor.

### Statistics

Data obtained through cell-based assays were evaluated using a two-tailed Students t-test. For RNA-seq, *p* values were converted to Benjamini-Hochberg *q*-values; only data with a False-Discovery Rate (FDR) less than 0.2 are reported.

## Results

We found previously that the α subunit of the G protein G_13_ increases the activity of one or more Gli transcription factors in C3H10T1/2 and pancreatic cancer cells [16]. Given that Smo relies heavily on these transcription factors in its actions, and that both Smo and G_13_ play important roles in a variety of developmental settings, we wanted to evaluate the potential for overlap in signaling between the two. We used C3H10T1/2 and 3T3-L1 cells as convenient models of osteogenic and adipogenic differentiation, respectively, and constitutively active forms of G_13_ and Smo, Gα_13_QL [27] and SmoA1 [23], to engage signaling.

**Fig 1:**
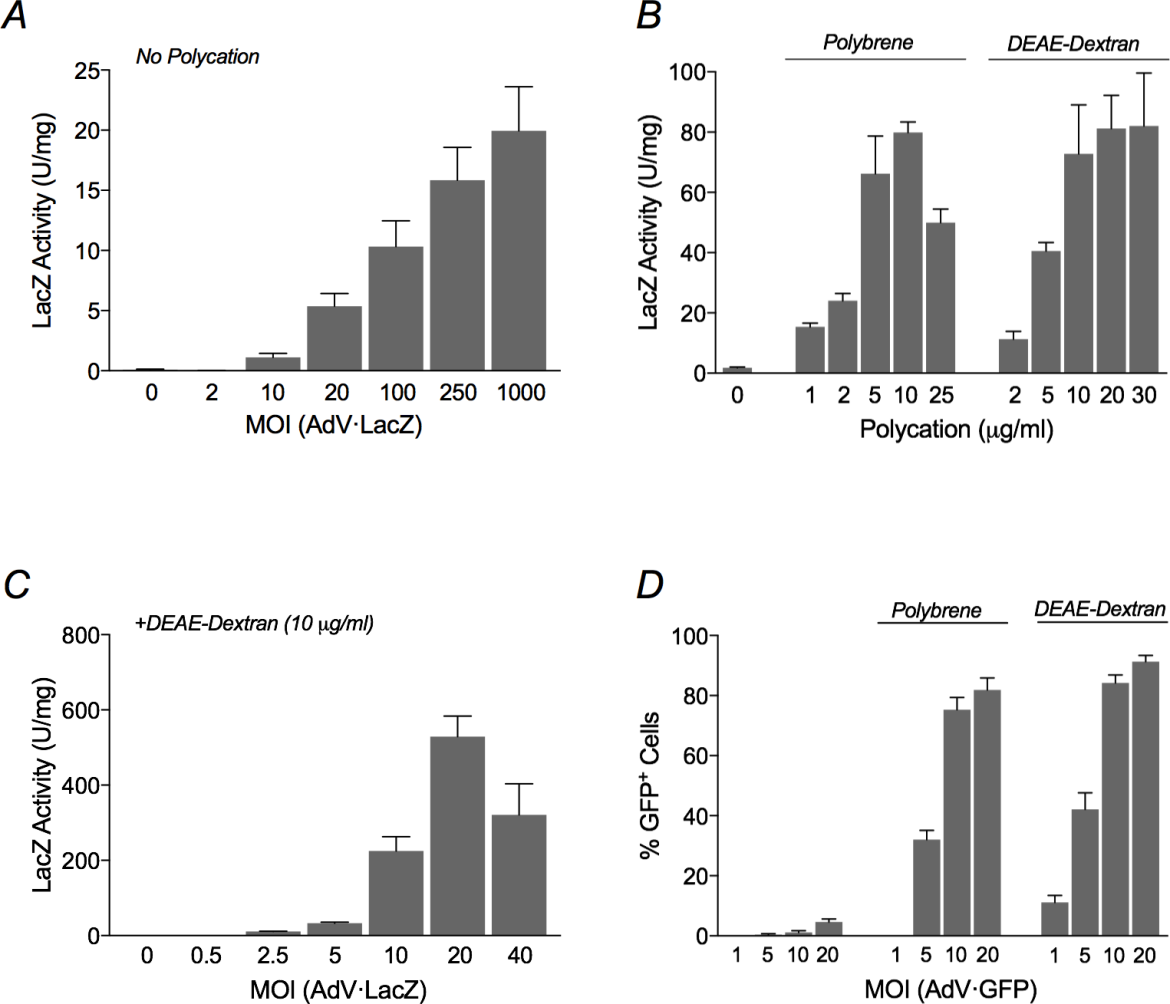
Enhancement of adenoviral transduction by polycations. C3H10T1/2 cells were transduced with recombinant adenoviruses expressing LacZ (AdV·LacZ) or GFP (AdV·GFP) in the presence or absence of polycations. (A) Cells were transduced with AdV·LacZ at the indicated MOIs in the absence of polycation. The cells were lysed 2 days later, and LacZ activity was measured. Activity is expressed as units per mg cell lysate. The data are averages of three different experiments, each carried out in triplicate, ± SEM; *p* <0 .05 for MOIs ≥ 20 relative to no virus. (B) Cells were transduced with AdV·LacZ at an MOI of 10 at the indicated concentrations of polybrene and DEAE-dextran. The cells were lysed 2 days later, and LacZ activity was measured. The data are from an individual experiment that represents three experiments total, each carried out in triplicate but using slightly different sets of concentrations of polycations, ± SEM; *p* < 0.01 at all concentrations of polycation relative to no polycation. (C) Cells were transduced with AdV·LacZ at the indicated MOIs in the presence of 10 μg/ml DEAE-Dextran. The cells were lysed 2 days later, and LacZ activity was measured. The data are averages of four different experiments, each carried out in triplicate, ± SEM; *p* < 0.05 for MOIs ≥ 2.5 relative to no virus. (D) Cells were transduced with AdV·GFP at the indicated MOIs without or with polybrene (5 μg/ml) or DEAE-dextran (10 μg/ml). The cells were fixed 2 days later, counterstained with DAPI, and evaluated as the number of GFP- relative to DAPI-positive cells by fluorescence microscopy. The data are from an individual experiment that represents three experiments total, each carried out using a minimum of 10 random fields for each data point, but using slightly different sets of concentrations of polycations, ± SEM; *p* < 0.01 for MOIs ≥ 5 in the presence of polycation relative to absence of polycation.

Our first challenge was to achieve a sufficiently good expression of SmoA1 and Gα_13_QL in C3H10T1/2 cells, as we had found these cells in previous studies to be quite difficult to transfect. We turned to recombinant adenoviruses. Pilot experiments with a recombinant adenovirus encoding LacZ revealed, still, only minimal expression following transduction of the cells using standard protocols, even at an MOI of 1000 (Fig 1, panel A; compare activities to panel C). We therefore explored a technique employing polycations, which are thought to facilitate attachment of adenoviruses to the negatively charged cell membrane absent other binding mechanisms [28]. We found that inclusion of polybrene or DEAE-dextran at the time of exposure of the cells to the recombinant adenoviruses increased levels of subsequent LacZ expression up to 40-fold, and did so at MOIs that were 50–100-fold lower (Fig 1B and 1C). Experiments with GFP, moreover, demonstrated that the polycations increased the percentage of cells expressing protein from 2–7% to about 80% (Fig 1D).

**Fig 2:**
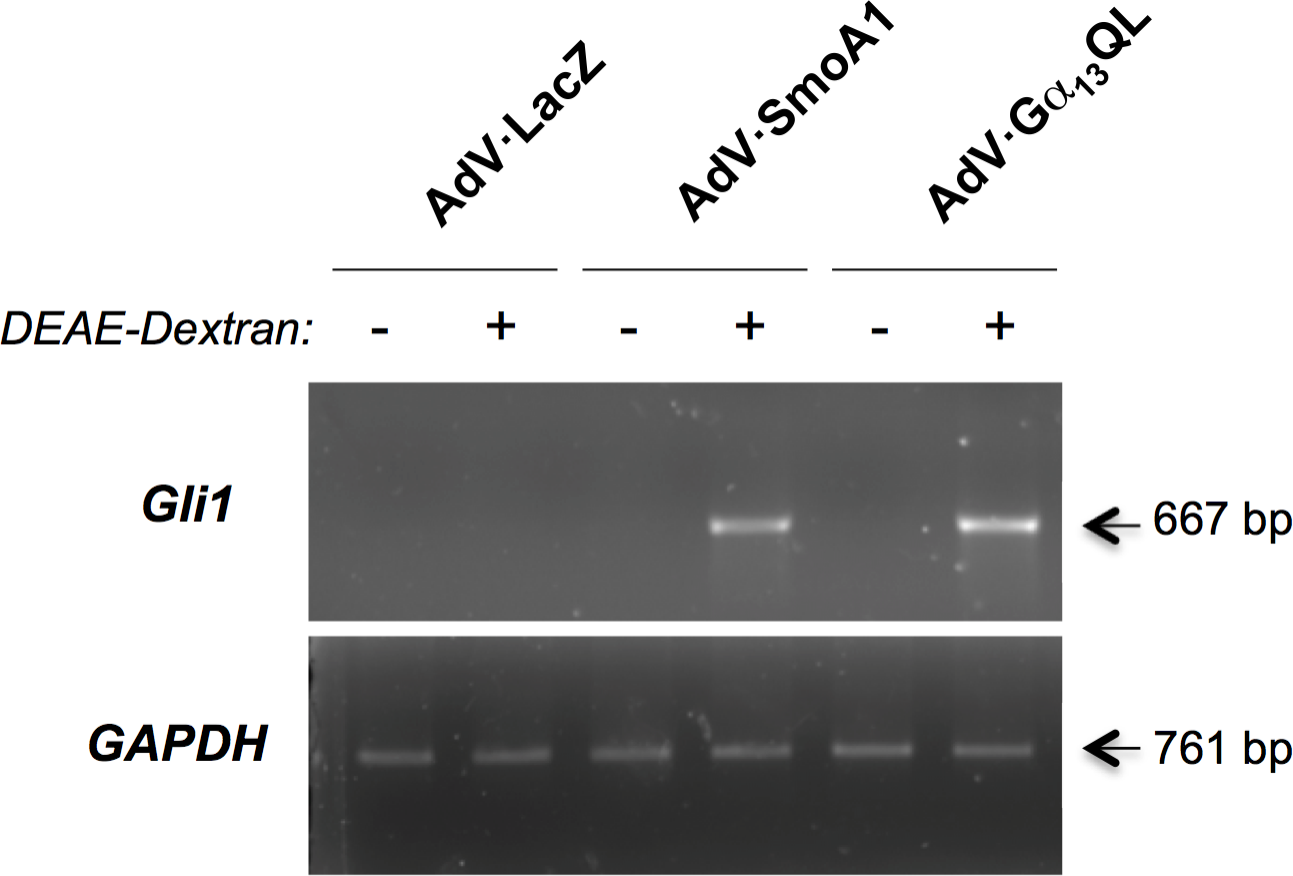
Induction of Gli1 by SmoA1 and Gα_13_QL. C3H10T1/2 cells were transduced with recombinant adenoviruses encoding LacZ, SmoA1, or Gα_13_QL (MOI = 10) in the presence or absence of DEAE-Dextran (10 μg/ml) as noted. At 2 days following transduction, RNA was extracted for PCR with primers specific for Gli1 and, as a control for loading, GAPDH. Arrows denote anticipated sizes of the products. Shown is one experiment, which is representative of three total.

We turned then to recombinant adenoviruses expressing SmoA1 and Gα_13_QL. Fig 2 shows that introduction of SmoA1 into C3H10T1/2 cells, now using DEAE-dextran, leads to an increase in Gli1 mRNA (Fig 2, see top panel). This is a well documented action of Smo, likely attributable to transactivation of *gli1* by Gli2 and/or Gli3 [29–31]. We found that Gα_13_QL also increases Gli1 mRNA. This finding could not have been predicted (see Discussion), but is consistent with the increase in Gli activity by Gα_13_QL observed previously [16].

**Fig 3:**
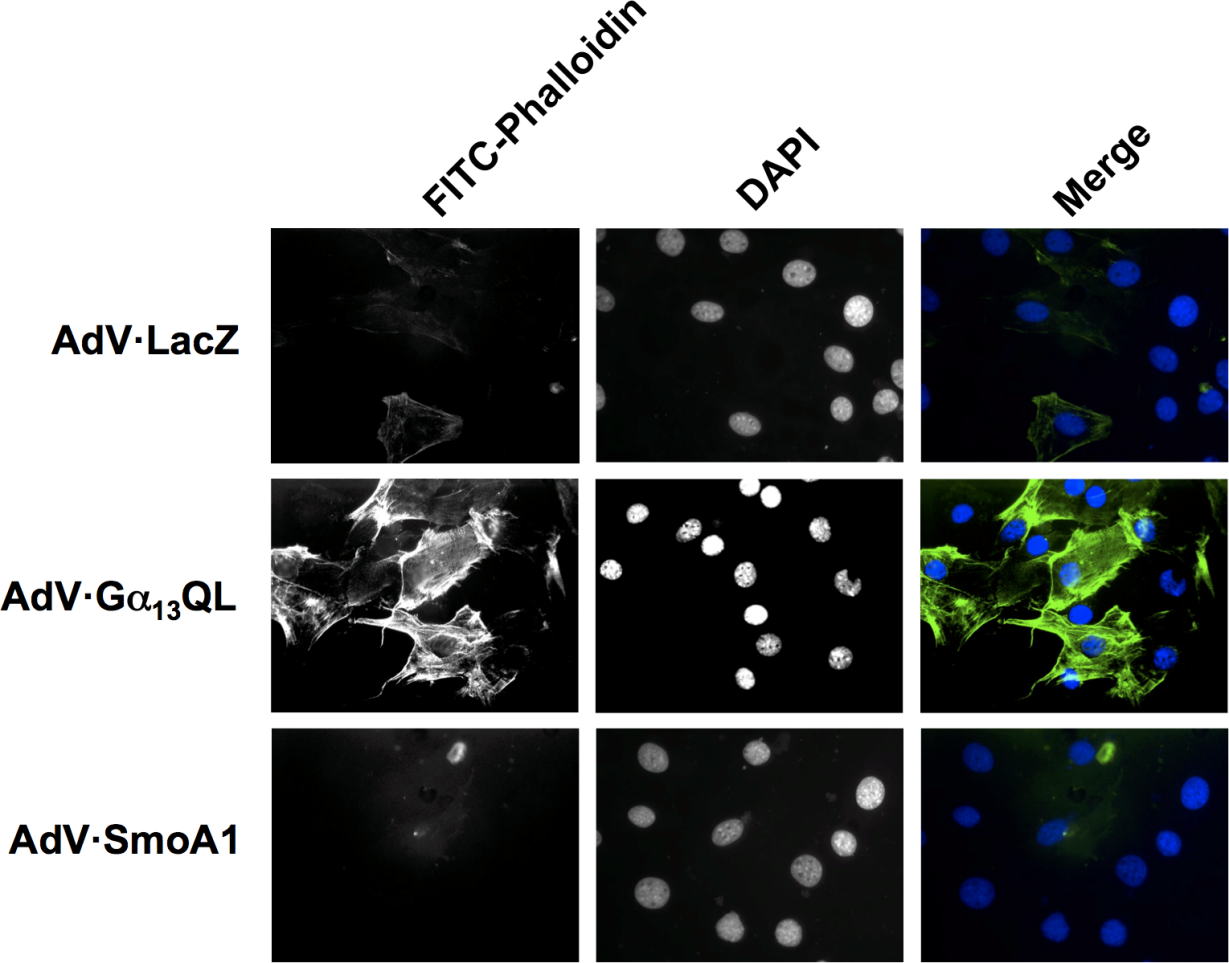
Stress fiber formation in response to SmoA1 and Gα_13_QL. C3H10T1/2 cells grown on coverslips were serum-starved for 12 h then incubated with recombinant adenoviruses encoding LacZ, SmoA1, or Gα_13_QL (MOI = 10) in the presence of DEAE-Dextran (10 μg/ml). They were serum-starved an additional 18 h and fixed with paraformaldehyde. F-actin was stained with FITC-Phalloidin, and nuclei were counterstained with DAPI. ‘Merge’ is pseudocolored. Shown is one experiment representative of three total. Few or no stress fibers were identified in the absence of transduction (S2 Fig).

While induction of Gli1 is a prototypic action of Smo, stress fiber formation is similarly a prototypic action of G_13_. The underlying mechanism is the activation through Gα_13_ of the monomeric G protein Rho [32]. Expression of Gα_13_QL in C3H10T1/2 cells causes, as anticipated, formation of actin fibers easily identified with FITC-phalloidin (Fig 3). Thus, SmoA1 (above) and Gα_13_QL (here) each engage in activities that attest to appropriate functionality. SmoA1 has no impact on FITC-phalloidin staining, at least under the constraints of the assay, e.g. expression and degree of cell confluency. While Gα_13_QL can mimic SmoA1 in increasing Gli1, therefore, SmoA1 does not to reciprocally mimic Gα_13_QL in causing stress fiber formation under the same conditions.

**Fig 4:**
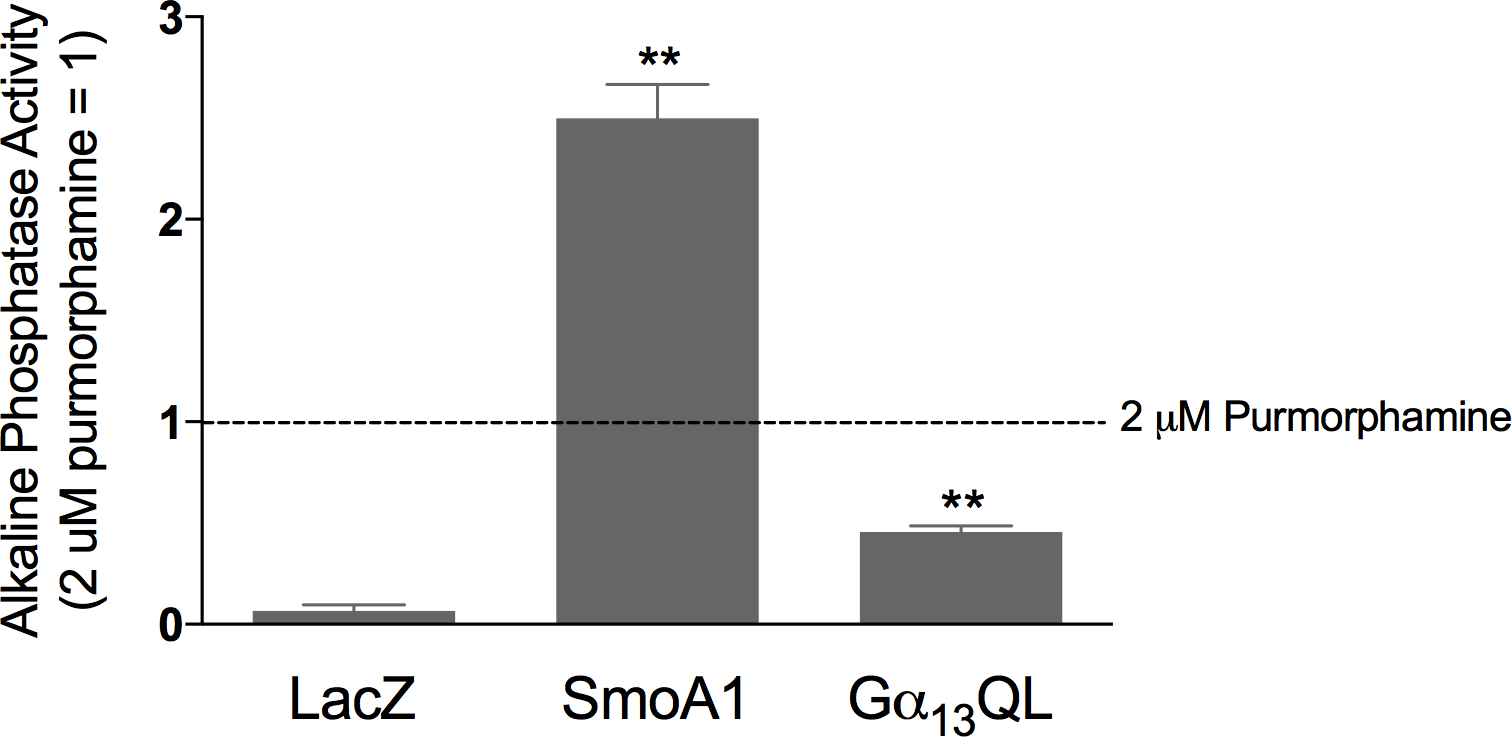
Alkaline phosphatase induction in response to SmoA1 and Gα_13_QL. C3H10T1/2 cells were transduced with recombinant adenoviruses encoding LacZ, SmoA1, or Gα_13_QL (MOI = 10) in the presence of DEAE-Dextran (10 μg/ml) then, together with cells exposed to 2 μM purmorphamine as a point of reference, cultured for 8 days in medium containing 50 μg/ml ascorbic acid and 10 mM β-glycerophosphate, at which point alkaline phosphatase activity was determined and normalized to that achieved with purmorphamine. The data are averages of 5 experiments, each performed in triplicate, ± SEM; **, p < 0.01 relative to LacZ.

Induction of alkaline phosphatase marks progression of C3H10T1/2 cells through osteoblastic differentiation [33–36]. The introduction of SmoA1 into these cells increases, as expected, alkaline phosphatase activity (Fig 4). The activity is greater than that achieved through endogenous Smo with purmorphamine, used as a point of reference. Gα_13_QL, too, increases alkaline phosphatase activity, however only modestly. We found the actions of Gα_13_QL on alkaline phosphatase, moreover, to require ascorbic acid and β-glycerophosphate (S3 Fig), which are components of a commonly used ‘pro-osteogenic’ media. The actions of SmoA1 did not require these reagents.

**Fig 5:**
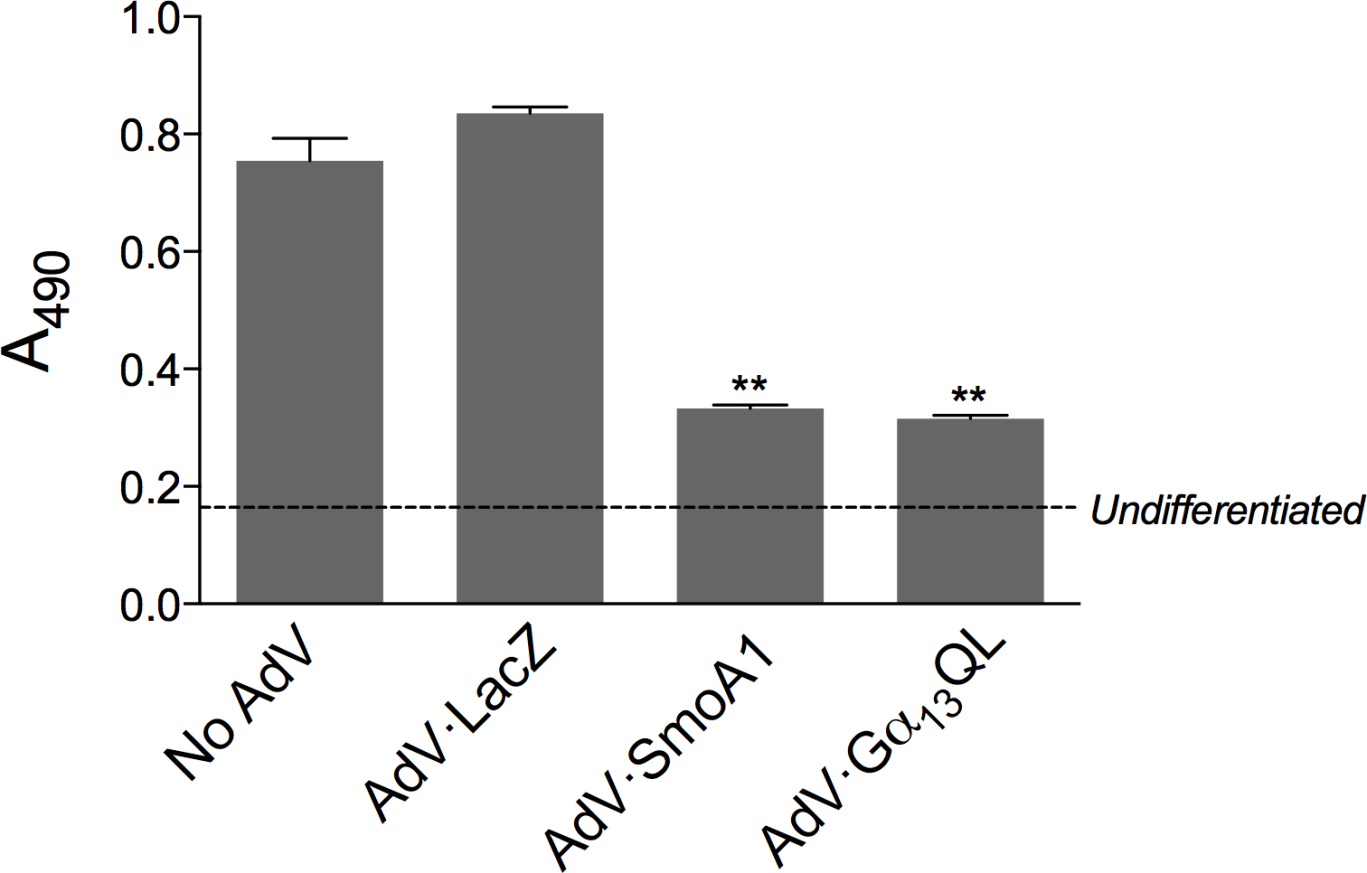
Effect of SmoA1 and Gα_13_QL on 3T3-L1 adipogenic differentiation. 3T3-L1 fibroblasts were transduced with recombinant adenoviruses encoding with LacZ, SmoA1, or Gα_13_QL (MOI of 10), or no virus, in the presence of DEAE-dextran (10 μg/ml). The medium was replaced, and the cells were induced to differentiate with a combination of IBMX (0.5 mM), dexamethasone (0.25 μM), rosiglitazone (2 μM), and insulin (1 μg/ml) as described in ‘Materials and Methods’. The cells were fixed at 2 weeks, and triglyceride accumulation was evaluated with Oil Red O. The data shown are from one experiment representative of three total, each carried out in triplicate, ± SEM; **, p < 0.01 relative to LacZ. ‘Undifferentiated’ refers to the absorbance for cells not induced to differentiate. For the three experiments, the average A_490_ for SmoA1 and Gα_13_QL relative to that for untransformed differentiated cells, subtracting the absorbance for undifferentiated cells, was 0.1 ± 0.07 for both SmoA1 and Gα_13_QL (p < 0.05).

Sonic hedgehog inhibits adipogenic differentiation [36,37]. To examine the effects of SmoA1 and Gα_13_QL on adipogenesis, we used mouse 3T3-L1 cells, which constitute a preadipocyte cell line that differentiates into adipocytes more easily and with greater efficiency than C3H10T1/2 cells. Fig 5 shows that both SmoA1 and Gα_13_QL inhibit adipogenesis. Specifically, they inhibit triglyceride accumulation as identified through Oil Red O staining of cells induced to differentiate with a combination of isobutylmethylxanthine, dexamethasone, insulin, and rosiglitazone. The degree of inhibition by the two proteins, about 80%, was equivalent.

We turned to RNA sequencing as a means to evaluate better the transcriptional events consequent to introduction of SmoA1 and Gα_13_QL. SmoA1 increased transcripts for approximately 6700 genes, while Gα_13_QL increased transcripts for 4900 genes. Of these, 3900 were common to the two proteins. The 100 genes most strongly induced are noted (S1 Table). Of the approximately 3800 and 4300 genes whose transcripts were decreased by SmoA1 and Gα_13_QL, about 3000 were common to the two. As shown (Table 1), changes in gene activity were consistent with the above cell assays: SmoA1 increased transcripts for Gli1 and three alkaline phosphatases, Akp3, Alpi, and Alppl2; Gα_13_QL increased transcripts for Gli1 and Akp3, with the increase in Akp3 being considerably less than that achieved by SmoA1; and SmoA1 and Gα_13_QL both decreased transcripts for several transcription factors relevant to adipogenesis.

**Table 1:** Changes in transcripts for selected genes in C3H10T1/2 cells following introduction of SmoA1 and Gα_13_QL, or exposure to purmorphamine.

		*SmoA1*	*Gα*_*13*_*QL*	*Purmorphamine*
	*-fold*			
***Immediate Hh signaling***				
	Gli1	2	7	277
***Alkaline phosphatases***				
	Akp3	4053	81	—
	Alpi	2527	—	—
	Alpl	—	—	607
	Alppl2	1220	—	—
***Adipogenesis***				
	c/EBPα	0.29	0.25	0.71
	c/EBPβ	0.54	0.29	—
	c/EBPδ	0.63	0.13	0.68
	PPARγ	0.69	—	0.87

Listed are *-fold* changes by SmoA1 and Gα_13_QL, relative to LacZ, and by 2 μM purmorphamine, relative to vehicle (0.02% DMSO), in transcripts from C3H10T1/2 cells of genes perceived relevant to the cell assays conducted in this study (n = 3 for SmoA1 and Gα_13_QL (vs. n = 3 for LacZ), n = 4 for purmorphamine (vs. n = 3 for vehicle), using changes in transcripts with an FDR < 0.2). ‘—‘, FDR ≥ 0.2, or transcripts not detected. Complete RNA-seq data are archived through GEO, specifically GSE98841 for SmoA1 and Gα_13_QL, and GSE111669 for purmorphamine.

The RNA-seq data utilizing recombinant adenoviruses revealed two anomalies. First, a BLAST analysis using a 60-nt portion of the SV40 polyA sequence immediately 3’ of the transgenes detected an average of 105, 1.7, and 29 reads for LacZ, SmoA1, and Gα_13_QL, respectively, normalized to 30 million reads total, indicating unequal transcription of the transgenes, despite being driven by the same promoter and not justified by the small differences noted in Materials and Methods in viral titer. Second, a high percentage of non-mouse reads, primarily adenovirus and including E1b and E3, were found for SmoA1 and Gα_13_QL samples (42% and 65%, respectively, versus 14% for LacZ). The presence of E1b and E3 would indicate some recombination to wildtype virus, however no cell lysis was evident in any of the above-mentioned assays.

Given the anomalies, and as well our desire to extend the studies to a transient setting, we turned to purmorphamine, a small-molecule agonist for Smo, which is endogenous to C3H10T1/2 cells [38–40]. Exposure of the cells to purmorphamine increased transcripts for about 4700 genes. Of these genes, about 1900, or 40%, matched those whose transcripts were increased by SmoA1. Of the approximately 3500 genes whose transcripts were decreased by purmorphamine, about 1400, or again 40%, matched those whose transcripts were decreased by SmoA1. Of the differences between purmorphamine and SmoA1, some were striking: the -fold increase in transcripts for Gli1 was 100 times greater with purmorphamine than that with SmoA1, and while SmoA1 increased transcripts for Akp3, Alpi, and Alppl2, purmorphamine increased transcripts for another phosphatase altogether, Alpl (Table 1). In an effort to filter out increases or decreases in transcripts for SmoA1 that were conceivably nonspecific, for instance relating to the adenovirus vector, we identified SmoA1 genes whose transcripts were uniquely changed in relation to Gα_13_QL then evaluated matches between these and the genes regulated by purmorphamine. Selection did not enrich for matches between the two. For genes whose transcripts were uniquely increased in SmoA1 relative to Gα_13_QL, 29% matched those whose transcripts were increased by purmorphamine, versus 28% without selection. For genes whose transcripts were uniquely decreased in SmoA1 relative to Gα_13_QL, 27% matched those whose transcripts were decreased by purmorphamine, versus 37% without selection. The lack of enrichment implies that the difference between SmoA1 and purmorphamine is not related to nonspecific events.

## Discussion

Our goal in this study was to begin to understand the extent to which G_13_ can replicate the actions of Smo. We had demonstrated previously that Gα_13_ activates Gli reporter constructs, but we wanted here to evaluate its actions in the context of differentiation, and of gene regulation expressly. We also came to a point of wanting to understand, in the context of Smo, differences in signaling between mutationally and agonist-activated receptor.

The choice of constitutively active forms of Smo and Gα_13_ was based on their ability to provide strong, unequivocal signals, and hence to provide a reasonable starting point in evaluating potential. Our choice of a receptor (SmoA1) vs. a G protein α subunit (Gα_13_QL) was dictated by the tools available. A constitutively active G_13_-selective receptor would have made sense, yet none exists. In our focus on G_13_, furthermore, we wanted to avoid G protein-independent forms of transduction.

We should not have been surprised at the poor efficiency of transduction for C3H10T1/2 cells: MOIs reported for this cell line are not uncommonly 100, and few studies report the percent of cells actually transduced [41–43]. The inclusion of polycations during exposure of the cells to recombinant adenoviruses, however, provided the efficiency we needed. Polycations are viewed to overcome charge repulsion between the negatively charged adenovirus particle and cell surface membrane [28], and can be advantageous if the cell lacks surface proteins utilized by the viruses as receptors for entry.

The increase in Gli1 mRNA promoted by Gα_13_QL in C3H10T1/2 cells, observed by PCR and verified by RNA-seq, while consistent with the increase in Gli activity previously determined by us for these cells [16], was not necessarily expected. We had found, specifically, in L3.6 pancreatic cancer cells that Gα_13_QL does not increase Gli1 despite increasing Gli reporter gene activity [16]. We suspect the difference between the two types of cells lies in Gli1 expression prior to introduction of Gα_13_QL. L3.6 cells express quite high levels of Gli1 at the outset, while C3H10T1/2 cells appear not to. It is conceivable, therefore, that the exact form of regulation, for example transcriptional (C3H10T1/2 cells) versus post-translational (L3.6 cells), is conditioned on preexisting levels of Gli1. We found the increase in Gli1 mRNA by Gα_13_QL in C3H10T1/2 cells to be comparable if not greater than that achieved with SmoA1. This has no bearing, however, on the specific activities of Gα_13_QL versus SmoA1. In fact, BLAST analysis of RNA-seq reads reveals that SmoA1 is considerably less well expressed than Gα_13_QL.

SmoA1 can activate the monomeric G protein Rho in some cells [6,44,45], but the lack of any effect of SmoA1 on stress fiber formation in C3H10T1/2 cells, at least under the conditions of the assay, suggests that it does not activate Rho in these cells. If the link between Smo and Rho is achieved through G_i_, as we and others have argued [6,44,46], then C3H10T1/2 cells may lack the mechanism through which G_i_ activates Rho. This is the case in the majority of cells [32]. The absence of stress fiber formation in response to SmoA1 helps to corroborate our assertion [16] that Smo does not couple to G_12_ or G_13_, as had been posited elsewhere [47], but again the argument is tempered by the constraints of the assay.

We find that Gα_13_QL, like SmoA1, can induce alkaline phosphatase activity, a marker of osteogenic differentiation. The induction is much less than that achieved by SmoA1. The difference in degree of induction is corroborated by RNA-seq: the three alkaline phosphatase genes most highly induced by SmoA1 are Akp3, Alpi, and Alppl2; Akp3 is induced by Gα_13_QL but not nearly to the same extent as by SmoA1. The increase in alkaline phosphatase activity achieved through Smo is normally credited to Gli1 [36,48]. While the increase in Gli1 mRNA by Gα_13_QL exceeds that achieved by SmoA1, this did not translate into a greater degree of alkaline phosphatase activity. It is conceivable that Gli1 is required but not sufficient and that Gα_13_QL does not engage the other requisite factors. Alternatively, the 2-day point of assay may not fully reflect increases in Gli1 by SmoA1 through the duration (8 days) of osteogenic induction.

Although SmoA1 and Gα_13_QL differ in the extent to which they induce alkaline phosphatase, the inhibition of adipogenesis by the two, as evaluated through triglyceride accumulation, is similar and substantial. Both moreover decreased C/EBPα, β, and δ, key transcription factors in adipogenesis [49] as evaluated by RNA-seq. SmoA1, but not Gα_13_QL, decreased PPARγ, another critical transcription factor. These data show a degree of overlap between SmoA1 and Gα_13_QL, but again with distinctions. It is important to bear in mind here, as for osteogenesis above, that differentiation is a slowly unfolding process and that gene transcripts are evaluated in the present study at only one, early time point.

RNA sequencing revealed considerable overlap in the total number of genes regulated by SmoA1 and Gα_13_QL. We harbored no expectations that LacZ, SmoA1, and Gα_13_QL would be expressed equally well, nor were they. The surprise was the low degree of expression in general, and of SmoA1 especially. Even at low levels of expression, however, specific and prototypic actions of the two proteins are easily discerned. Viral recombination was a possible issue, but the above-mentioned prototypic actions helped to allay this concern, and as well the fact that there was no evidence of morphological abnormalities even up to 2 weeks following transduction.

We utilized purmorphamine subsequently with the intent of confirming some of the targets identified with SmoA1. While some were of course identified, we were surprised by the degree of mismatch. Only 40% of the genes up- or down-regulated by purmorphamine match those similarly up- or down-regulated by SmoA1. This does *not* negate the SmoA1 data, which are in accord with expectations. Some differences, for example that relating to Gli1, might be attributable to the above-mentioned low expression of SmoA1 relative to endogenous Smo. However, the differential regulation of alkaline phosphatases begs additional discussion. First, especially given the role of Gli1 in morphogenic signaling, it is possible that the differences in levels of Gli1 expression are translated differently by individual target genes. Second, as subcellular positioning is a critical aspect to some forms of Smo signaling [50–53], it is conceivable that an already activated form of Smo produced from a vector targets differently from that of endogenous Smo. Third, there may exist bias in signaling between the conformations of mutationally versus agonist-activated forms of Smo. The differences in signaling are likely not due to viral burden or the above-mentioned possibility of viral recombination. The percent regulated genes shared between SmoA1 and purmorphamine remain the same or decrease when selecting for those unique to SmoA1 relative to Gα_13_QL.

The use of constitutively active proteins is normally made with the intent of establishing a framework for potential downstream pathways, whether the constitutively active proteins occur in nature, as can those of Smo, or more transient forms of signaling are engaged. Potential in the case of the latter is, of course, not always realized. The comparison between SmoA1 and purmorphamine underscores this point. While overt cellular actions might be similar, underlying molecular events need not be. Our work does not negate the use of comparisons among constitutively active proteins in establishing a framework, but suggests that the concept of a framework must be reworked as precision at the molecular level increases.

## Supporting information

S1 Fig Alkaline phosphatase expression as a function of purmorphamine concentration. (DOCX)

S2 Fig Few if any stress fibers exist in the absence of transduction. (PDF)

S3 Fig Alkaline phosphatase activity following introduction of Gα13QL depends on the nature of the medium. (DOCX)

S1 Table Genes exhibiting the greatest -fold increases in transcripts following introduction of SmoA1 and Gα13QL, relative to LacZ, in C3H10T1/2 cells. (DOCX)
