# Supplementary material for: Overlap in signaling between Smoothened and the α subunit of the heterotrimeric G protein G13
Source: PLoS One. 2018 May 15;13(5):e0197442. doi: 10.1371/journal.pone.0197442 (PMC5953476; doi:10.1371/journal.pone.0197442)
Supplement: S1 Fig — (DOCX) [file pone.0197442.s001.docx]

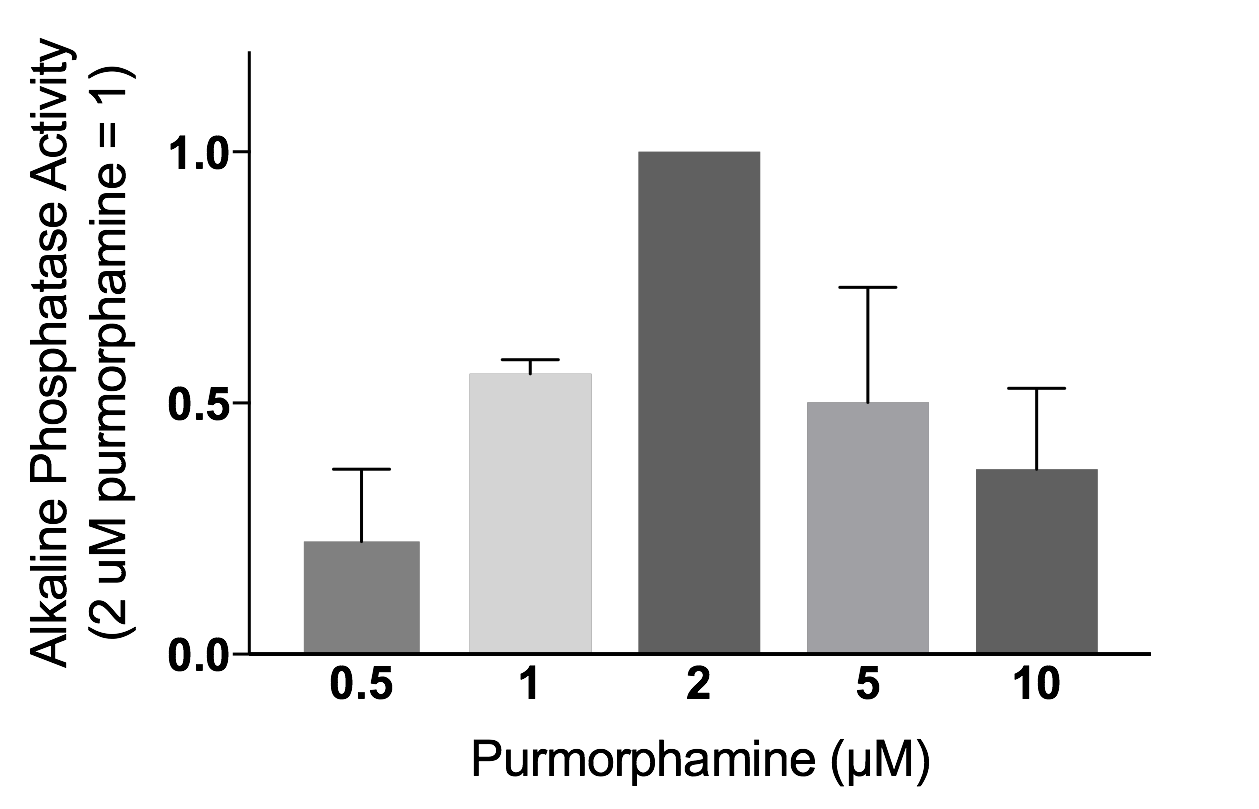


**S1 Figure. Alkaline phosphatase expression as a function of purmorphamine concentration.** C3H10T1/2 cells were treated with various concentrations of purmorphamine for 5–7 days, at which point alkaline phosphatase activity was determined. The data represent an average of 2–5 experiments, each performed in duplicate or triplicate and normalized to 2 uM purmorphamine, ±SEM.
