# Supplementary material for: Overlap in signaling between Smoothened and the α subunit of the heterotrimeric G protein G13
Source: PLoS One. 2018 May 15;13(5):e0197442. doi: 10.1371/journal.pone.0197442 (PMC5953476; doi:10.1371/journal.pone.0197442)

S2 Figure. Few if any stress fibers exist in the absence of transduction. C3H10T1/2 cells treated with or without AdV·Gα_{12}QL, used here (rather that AdV·Gα_{13}QL) as a positive control, were fixed and stained with FITC-phalloidin and counterstained with DAPI as described in 'Materials and methods' of the manuscript. The 'merge' is pseudocolored. The lack of staining without transduction was noted as well in several other experiments with other positive controls and, of course, for AdV·LacZ.

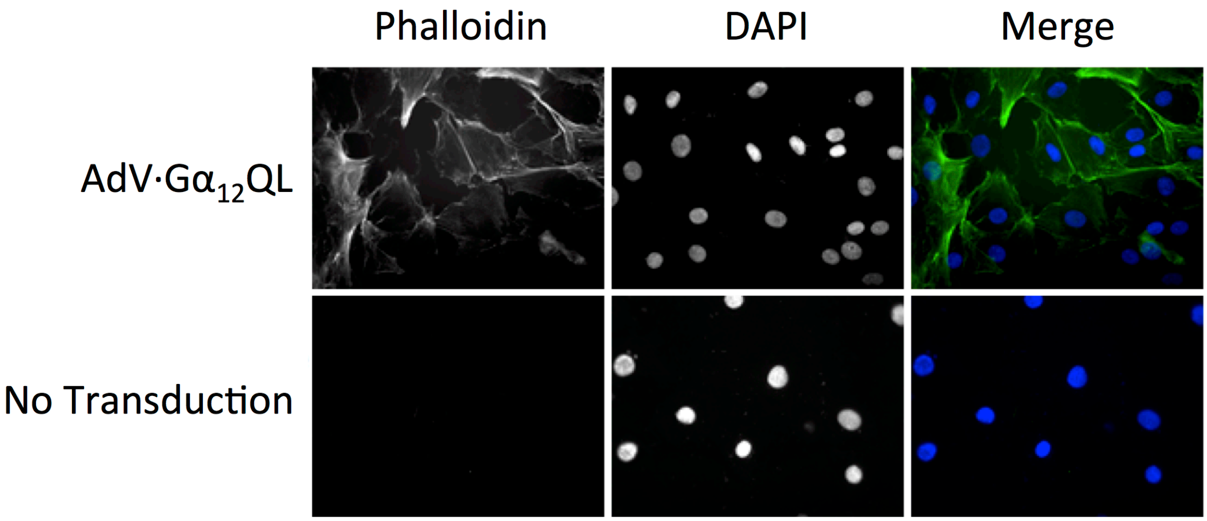

Supplement: S2 Fig — (PDF) [file pone.0197442.s002.pdf]
