# Supplementary material for: Overlap in signaling between Smoothened and the α subunit of the heterotrimeric G protein G13
Source: PLoS One. 2018 May 15;13(5):e0197442. doi: 10.1371/journal.pone.0197442 (PMC5953476; doi:10.1371/journal.pone.0197442)

S3 Figure. **Alkaline phosphatase activity following introduction of Gα_13_QL depends on the nature of the medium.** C3H10T1/2 cells transduced with recombinant adenovirus encoding Gα_13_QL were cultured for 8 days in medium without (non-osteogenic) or with (osteogenic) 50 μg/ml ascorbic acid and 10 mM β-glycerophosphate, at which point alkaline phosphatase activity was determined and normalized to that achieved with 2 μM purmorphamine. The data represent 3 and 5 experiments, respectively, for non-osteogenic and osteogenic medium in triplicate, ± SEM; **, p < 0.01 relative to 0. Data for osteogenic medium and 2 μM purmorphamine are the same as used in Figure 4 of the manuscript.


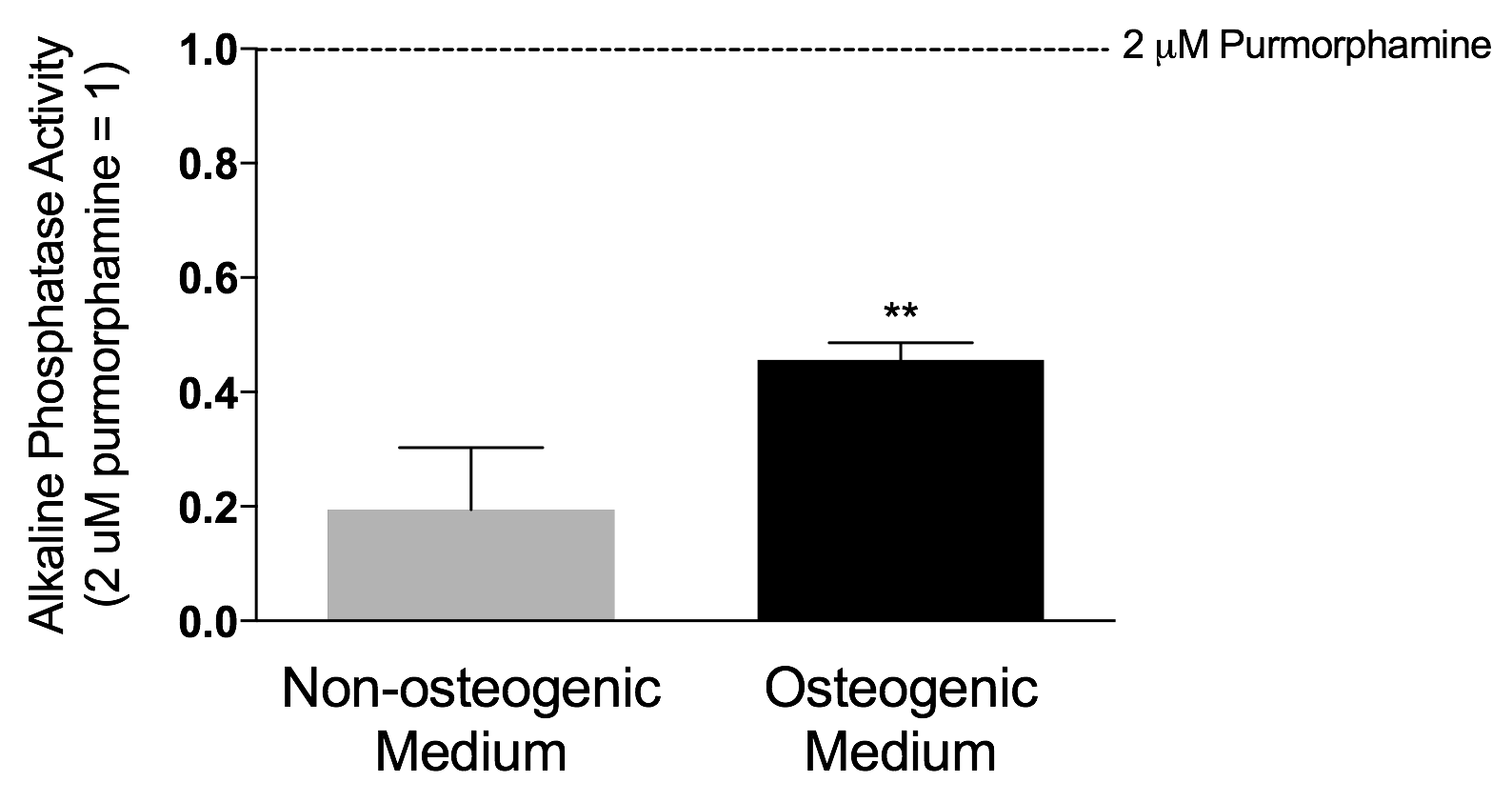

Supplement: S3 Fig — (DOCX) [file pone.0197442.s003.docx]
