# Supplementary material for: Overlap in signaling between Smoothened and the α subunit of the heterotrimeric G protein G13
Source: PLoS One. 2018 May 15;13(5):e0197442. doi: 10.1371/journal.pone.0197442 (PMC5953476; doi:10.1371/journal.pone.0197442)
Supplement: S1 Table — (DOCX) [file pone.0197442.s004.docx]

**S1 Table: Genes exhibiting the greatest -fold increases in transcripts following introduction of SmoA1 and Gα_13_QL, relative to LacZ, in C3H10T1/2 cells.** Increases in transcripts two days following transduction were determined by RNA-sequencing as described in ‘Materials and methods’ of the manuscript. Genes common to the two lists are highlighted in bold font.

**Rank SmoA1 Gα_13_QL**

*Gene -fold Gene -fold*

*increase increase*

| 1 |  | Akp3 | 4053 |  | **Zdbf2** | 922 |
| --- | --- | --- | --- | --- | --- | --- |
| 2 |  | Alpi | 2527 |  | **Tmem40** | 810 |
| 3 |  | Alppl2 | 1220 |  | Ryr1 | 807 |
| 4 |  | **Obox6** | 447 |  | **Diras1** | 658 |
| 5 |  | **Vil1** | 413 |  | **1300017J02Rik** | 547 |
| 6 |  | **Zdbf2** | 391 |  | Lct | 506 |
| 7 |  | **Il17f** | 374 |  | **Dyrk4** | 503 |
| 8 |  | **Tmem40** | 364 |  | Grin1 | 503 |
| 9 |  | **1300017J02Rik** | 329 |  | Fam23a | 503 |
| 10 |  | Muc2 | 273 |  | Tnxb | 487 |
| 11 |  | **Cntn2** | 262 |  | **Vil1** | 466 |
| 12 |  | Tcfl5 | 247 |  | **Atp8b3** | 456 |
| 13 |  | **Lingo3** | 237 |  | **Il17f** | 400 |
| 14 |  | **Diras1** | 203 |  | **Obox6** | 389 |
| 15 |  | **Obscn** | 201 |  | Mmp9 | 350 |
| 16 |  | **Spdef** | 198 |  | **Ccdc125** | 343 |
| 17 |  | **Rph3a** | 195 |  | **Hkdc1** | 337 |
| 18 |  | Dnahc17 | 192 |  | Tmem63c | 304 |
| 19 |  | **Atcay** | 191 |  | **Lingo3** | 276 |
| 20 |  | **Rab3b** | 186 |  | **Aqp3** | 260 |
| 21 |  | **Dyrk4** | 182 |  | Asgr1 | 258 |
| 22 |  | **Cpne5** | 179 |  | Scg5 | 254 |
| 23 |  | **Neurl3** | 177 |  | Gal3st1 | 253 |
| 24 |  | **Msh4** | 168 |  | **Itgam** | 232 |
| 25 |  | **Cplx1** | 160 |  | **Msh4** | 226 |
| 26 |  | **Pigr** | 160 |  | Cyp3a57 | 225 |
| 27 |  | **Crx** | 152 |  | **Atcay** | 223 |
| 28 |  | **Pdzk1** | 151 |  | **Allc** | 219 |
| 29 |  | **Itgam** | 145 |  | Gabrr2 | 217 |
| 30 |  | Psd2 | 144 |  | **Mgat5b** | 215 |
| 31 |  | **Ccdc125** | 143 |  | Tcfcp2l1 | 205 |
| 32 |  | **Rad21l** | 136 |  | **Cpne5** | 203 |
| 33 |  | **Mgat5b** | 135 |  | **Rph3a** | 203 |
| 34 |  | St6galnac2 | 132 |  | **Cyp2c55** | 202 |
| 35 |  | **Stmn3** | 129 |  | Pygm | 197 |
| 36 |  | **Nlrp6** | 123 |  | **Spdef** | 197 |
| 37 |  | Spesp1 | 123 |  | **Tnnc2** | 196 |
| 38 |  | **Atp8b3** | 123 |  | **Pdzk1** | 196 |
| 39 |  | **BC052486** | 122 |  | **Obscn** | 190 |
| 40 |  | Stox1 | 120 |  | Myh15 | 189 |
| 41 |  | **Ntsr1** | 119 |  | Grk1 | 178 |
| 42 |  | **Cyp2c55** | 117 |  | Jakmip3 | 174 |
| 43 |  | **Aqp3** | 116 |  | **BC013712** | 173 |
| 44 |  | **Cnr2** | 113 |  | **Rab3b** | 172 |
| 45 |  | **Hkdc1** | 109 |  | **Ghrl** | 172 |
| 46 |  | **Tnnc2** | 109 |  | **Htr5b** | 171 |
| 47 |  | **Tex21** | 109 |  | **Ccdc11** | 170 |
| 48 |  | **Myh3** | 108 |  | **Nlrp6** | 170 |
| 49 |  | **Gm15319** | 108 |  | **Ak7** | 170 |
| 50 |  | **Gm884** | 108 |  | **Cntn2** | 168 |
| 51 |  | Mb | 107 |  | **Rad21l** | 167 |
| 52 |  | RP23-27D5.4 | 107 |  | Myt1 | 165 |
| 53 |  | **Trank1** | 105 |  | Colec11 | 162 |
| 54 |  | Mgl2 | 102 |  | Epha8 | 156 |
| 55 |  | **Allc** | 101 |  | **Cdh22** | 155 |
| 56 |  | **BC125332** | 98 |  | **Pigr** | 154 |
| 57 |  | **Ccdc11** | 98 |  | **Trank1** | 152 |
| 58 |  | **Ghrl** | 97 |  | 4930447C04Rik | 151 |
| 59 |  | **Ak7** | 97 |  | D630003M21Rik | 151 |
| 60 |  | Ripk4 | 96 |  | Myo15 | 147 |
| 61 |  | **Pecam1** | 94 |  | Slc17a8 | 146 |
| 62 |  | Gm595 | 94 |  | **BC052486** | 143 |
| 63 |  | **Gm6588** | 94 |  | **Asgr2** | 142 |
| 64 |  | Aldh8a1 | 94 |  | Tgm3 | 142 |
| 65 |  | **4930444P10Rik** | 93 |  | Evpl | 142 |
| 66 |  | **Htr5b** | 92 |  | Sag | 142 |
| 67 |  | **Asgr2** | 91 |  | **Neurl3** | 136 |
| 68 |  | **Lrrc67** | 91 |  | **Tdrd6** | 136 |
| 69 |  | Fam101a | 91 |  | **4930444P10Rik** | 135 |
| 70 |  | **Ampd1** | 90 |  | **Gjc2** | 132 |
| 71 |  | **Cdh22** | 90 |  | Tmem30c | 132 |
| 72 |  | Nrip3 | 87 |  | **Gm884** | 131 |
| 73 |  | Klf17 | 85 |  | **Stoml3** | 130 |
| 74 |  | **Stoml3** | 85 |  | **Cplx1** | 130 |
| 75 |  | 4933406J08Rik | 84 |  | **Pecam1** | 130 |
| 76 |  | Kncn | 84 |  | **Gm15319** | 129 |
| 77 |  | Myo16 | 84 |  | Syce2 | 129 |
| 78 |  | Gm10827 | 83 |  | **Lrrc67** | 128 |
| 79 |  | Gm10332 | 83 |  | Lck | 126 |
| 80 |  | **Lefty2** | 82 |  | **Stmn3** | 126 |
| 81 |  | Gpx5 | 82 |  | **Gm6588** | 124 |
| 82 |  | **4931423N10Rik** | 82 |  | Zfp811 | 123 |
| 83 |  | Rsph6a | 82 |  | Dnaic2 | 123 |
| 84 |  | Rundc3b | 81 |  | **Ntsr1** | 122 |
| 85 |  | 5830462I19Rik | 81 |  | **Crx** | 121 |
| 86 |  | **BC013712** | 80 |  | **4931423N10Rik** | 120 |
| 87 |  | 2310050B05Rik | 79 |  | Rfx4 | 119 |
| 88 |  | Nup210 | 78 |  | **Ampd1** | 119 |
| 89 |  | Hnf4a | 76 |  | **BC125332** | 117 |
| 90 |  | **Gjc2** | 76 |  | **Fam123c** | 117 |
| 91 |  | **Fam123c** | 75 |  | **Oprd1** | 116 |
| 92 |  | Mansc1 | 75 |  | **Cnr2** | 116 |
| 93 |  | Gm12630 | 74 |  | Apom | 116 |
| 94 |  | 3110035E14Rik | 74 |  | **Lefty2** | 113 |
| 95 |  | **Oprd1** | 74 |  | Nfasc | 113 |
| 96 |  | **Tdrd6** | 74 |  | Gm4840 | 112 |
| 97 |  | 4930407I10Rik | 71 |  | **Tex21** | 111 |
| 98 |  | Cyp2c65 | 70 |  | Col6a4 | 110 |
| 99 |  | Itgb1bp3 | 70 |  | 1700109H08Rik | 110 |
| 100 |  | Ucp1 | 70 |  | Plch2 | 109 |
